# Evaluation of 16S rRNA Hypervariable Regions for Bioweapon Species Detection by Massively Parallel Sequencing

**DOI:** 10.1155/2020/8865520

**Published:** 2020-09-26

**Authors:** Victor H. G. Dias, Priscila da S. F. C. Gomes, Allan C. Azevedo-Martins, Bianca C. A. Cabral, August E. Woerner, Bruce Budowle, Rodrigo S. Moura-Neto, Rosane Silva

**Affiliations:** ^1^Instituto de Biofísica Carlos Chagas Filho, Universidade Federal do Rio de Janeiro, Rio de Janeiro, Brazil; ^2^Center for Human Identification, University of North Texas Health Science Center, Fort Worth, Texas 76107, USA; ^3^Instituto de Biologia, Universidade Federal do Rio de Janeiro, Rio de Janeiro, Brazil

## Abstract

Molecular detection and classification of the bacterial groups in a sample are relevant in several areas, including medical research and forensics. Sanger sequencing of the 16S rRNA gene is considered the gold standard for microbial phylogenetic analysis. However, the development of massively parallel sequencing (MPS) offers enhanced sensitivity and specificity for microbiological analyses. In addition, 16S rRNA target amplification followed by MPS facilitates the combined use of multiple markers/regions, better discrimination of sample background, and higher sample throughput. We designed a novel set of 16S rRNA gene primers for detection of bacterial species associated with clinical, bioweapon, and biohazards microorganisms via alignment of 364 sequences representing 19 bacterial species and strains relevant to medical and forensics applications. In silico results indicated that the hypervariable regions (V1V2), (V4V5), and (V6V7V8) support the resolution of a selected group of bacteria. Interspecies and intraspecies comparisons showed 74.23%–85.51% and 94.48%–99.98% sequencing variation among species and strains, respectively. Sequence reads from a simulated scenario of bacterial species mapped to each of the three hypervariable regions of the respective species with different affinities. The minimum limit of detection was achieved using two different MPS platforms. This protocol can be used to detect or monitor as low as 2,000 genome equivalents of bacterial species associated with clinical, bioweapon, and biohazard microorganisms and potentially can distinguish natural outbreaks of pathogenic microorganisms from those occurring by intentional release.

## 1. Introduction

Bioterrorism is defined as the intentional and planned release of pathogens or toxins targeting humans, animals, plants, or materials. Bioterrorists can use biological agents to promote epidemics, create fear and panic in the population, overload health systems, and impact the economy, and they may be motivated by political or ideological reasons [1, 2]. Techniques for the detection of microorganisms of biodefense interest should be as sensitive and specific as possible, without interference from external contaminants (i.e., be robust), to minimize false-positives and false-negatives. Furthermore, it is preferable for these detection techniques to be easy to operate, provide rapid results, and have a large enough throughput capacity to analyze multiple samples and target pathogens simultaneously. Recent developments in molecular biology technologies have reduced the reliance on culture-based methods, which are selective and time-consuming [3]. In this regard, the development of massively parallel sequencing (MPS) has facilitated microbiological analyses, such as in forensic applications. The desktop versions of MPS technology have a throughput of 100 million to 15 billion bases per run. Therefore, with targeted sequencing, the same region of DNA can be sequenced many times, giving a greater read depth, which in turn provides operators with greater confidence in the results. With target enrichment methods, such as polymerase chain reaction amplification, positive results can be obtained from samples containing low quantities of DNA [4]. Specific gene target strategies, such as 16S rRNA, make it easy to assess the same gene across different organisms. The inclusion of MPS next generation sequencing (NGS) platforms for 16S rRNA increased the capacity of the identification of the bacterial members of microbiome communities by several orders of magnitude at a reduced cost. In addition, since only a short amplicon is sequenced, much higher coverage is obtained. Nevertheless, one drawback of this approach that typically targets only one segment of 16S rRNA is the inability to provide resolution to the genus level because of the shorter sequence length (60 nucleotides) and higher error rates [5]. Longer reads are generally accurate up to the genus level. Another aspect for resolution is the choice of the nine hypervariable regions (V1–V9) within the 16S rRNA gene. Several authors have assessed the efficiency of using different combinations of the hypervariable regions [6–8]; however, selection of the regions has been dependent on published or in-house-designed protocols, rather than the nature of the samples of interest [9, 10]. Usually, after sample processing, the generated data are compared to a database to facilitate taxa identification [11]. Hugenholtz et al. [12] showed that two or more 16S rRNA gene hypervariable regions could provide the phylogenetic division of microorganisms into monophyletic groups depending on the reference database and the different choices of classification [13]. Nevertheless, microbial composition data differ depending on the primers and sequencing platforms used [14]. Chakravorty et al. [15] analyzed V3 and V6 region sequences from 110 bacteria that infect humans, including 11 considered to be potential biological weapons. Their analysis suggested that the V6 region is the best choice for distinguishing between these 11 bacterial species of concern, except for *Escherichia sp*. and *Salmonella sp*., which are closely related genera. In addition, the target sequence size of less than 500 bp may limit resolution when only using the 16S rRNA gene, as many environmental species in the current databases may be homologous at the same portion of the gene. There are commercial kits for 16S rRNA gene metagenomic analysis [16] containing PCR primers for only the V3 and V4 regions. These primers, however, may not provide the highest possible resolution for 16S rRNA gene amplification [17]. In addition, commercially available 16S rRNA panels for MPS target areas of the gene for clinical purposes again not necessarily provide the desired resolution. Regardless, panels should enable the detection of bacteria in samples with low microbial density or those contaminated by host DNA, such as human tissue and low biomass samples, that are particularly susceptible to bias [10]. Currently, the combined 16S rRNA gene and MPS analysis is not optimized for biocrime application or the continuous surveillance and tracking of emerging infectious diseases at the genus/species level [18]. Our study presents a novel set of primers for the 16S rRNA gene that, combined with MPS, results in a sensitive and specific tool for the rapid identification of selected bacterial groups of biothreat and microbial forensic interest. Here, we demonstrate the use of three pairs of primers targeting the combination of three hypervariable regions of the 16S rRNA gene that successfully quantified and identified a specific group of microorganisms in complex samples using mock communities with human or environmental backgrounds. Furthermore, the use of mock samples allowed us to measure the sensitivity of this procedure for distinguishing cases of natural occurring pathogenic microorganisms from intentionally released biological agents.

## 2. Materials and Methods

### 2.1. Primer Design of 16S rRNA Gene Hypervariable Region

A total of 12,632 nucleotide sequences of the 16S rRNA gene were downloaded from the Greengenes databank [19]. We selected species and strains of the 19 most pathogenic bacteria considered to be of microbial forensic interest by the Center for Disease Control and Prevention (USA), which are *Bacillus anthracis*, *Brucella abortus*, *Brucella melitensis*, *Brucella suis*, *Burkholderia mallei*, *Burkholderia pseudomallei*, *Clostridium botulinum*, *Clostridium perfringens*, *Coxiella burnetii*, *Escherichia coli*, *Francisella tularensis*, *Rickettsia prowazekii*, *Salmonella enterica*, *Shigella boydii*, *Shigella dysenteriae*, *Shigella flexneri*, *Shigella sonnei*, *Vibrio cholerae*, and *Yersinia pestis*. Duplicated sequences and those below 1,200 bp in length were removed. The remaining 364 sequences (Table S1) were aligned to generate a consensus sequence for the 16S rRNA gene variable regions (V1–V9 sequences). Alignments were performed using ClustalW plugin [20] and CLC Genomic Workbench Software, version 8.5 (Qiagen, Hilden, Germany). The obtained 16S rRNA gene consensus sequence from each of the 19 species was used to assess its potential use for distinguishing among all the selected bacterial species. Universal primers were designed using Primer 3 2.3.4 plugin [21] of the Geneious™ 8.1.3 package (Biomatters Limited, Auckland, New Zealand) with the following criteria: product size adjusted for 300–400 bases; optimal product size of 350 bases; external to the target region (variable regions) and a maximum of four degeneracies; primer size of 18–27 bases; 50% GC; and optimal melting temperature (Tm) of 60°C.

### 2.2. Phylogenetic Tree Construction

The software MEGA, version 6.0 [22], was used to generate the phylogenetic trees using the maximum likelihood (ML) method and the Kimura-2 substitution model with gamma substitution and bootstrap of 1,000 replicates.

### 2.3. Human and Environmental DNA Samples

A human DNA sample was isolated from the peripheral blood of a healthy individual with informed consent (project approved by the Brazilian Research Ethics Committee, no. 536/10), following a standard protocol [23]. The sample was anonymized before analysis. The environmental DNA sample was obtained from the water reservoir of the Samuel hydroelectric plant, Rondônia, Brazil. DNA isolation of the lyophilized environmental sample was performed using PowerSoil™ DNA Isolation Kit (Qiagen, Hilden, Germany), as previously described [24]. Previous analysis of this environmental DNA sample showed that 98% of the DNA in the sample was bacterial [24].

### 2.4. DNA Mock Communities

Mock communities of bacterial DNA were constructed using genome equivalents (GE) to test the efficacy of the 16S rRNA gene primers panel. The GE for each organism was calculated as 01 GE = (Genome size (in bp) × 660 g)/6.02 × 10^23^ bp. Three mock communities were generated: [1] mock community containing 2,000 GE DNA from 20 bacterial species (readily accessible for this study), *B*. *anthracis*, *Burkholderia cepacia*, *Bacillus cereus*, *Clostridium difficile*, *C*. *perfringens*, *E*. *coli*, *Klebsiella pneumoniae*, *Mycobacterium tuberculosis*, *Neisseria meningitidis*, *Proteus mirabilis*, *Staphylococcus aureus*, *S*. *enterica*, *S*. *flexneri*, *S*. *sonnei*, *Staphylococcus epidermidis*, *V*. *cholerae*, *Yersinia enterocolitica*, *Y*. *pestis*, *Achromobacter xylosoxidans*, and *Propionibacterium acnes* (Supplemental Table S2); [2] mock community scenarios of variable GE (5 GE, 100 GE, or 2,000 GE) of *E*. *coli*, *V*. *cholerae*, and *S*. *enterica* (Supplemental Tables S2 and S3) were prepared for the detection of 16S rRNA gene using human gDNA as background (30 ng); [3] mock community of *E*. *coli*, *V*. *cholerae*, and *S*. *enterica* (0 GE and 2,000 GE) using environmental DNA (30 ng) (Tables S2 and S3).

### 2.5. Target Amplification

Amplification reactions were prepared separately, in duplicate, for each pair of primers (16S rRNA gene regions (V1V2), (V4V5), and (V6V7V8)) in a volume of 50 *μ*l, containing 0.4 *μ*M of each primer, 0.1 mM dNTPs (dATP, dTTP, dCTP, and dGTP) (Thermo Fisher Scientific, Waltham, USA), 1.5 mM MgCl_2_, 2.5 units of Platinum Taq DNA Polymerase (Invitrogen, Carlsbad, USA), 1 × polymerase buffer (Invitrogen, Carlsbad, USA), and 30 ng of DNA. Alternatively, amplification reactions were prepared using the Multiplex PCR Plus Kit (Qiagen, Hilden, Germany), following the manufactured protocol. The amplification conditions were 94°C for 5 minutes, followed by 17 cycles of denaturation at 94°C for 30 seconds, annealing at 55°C for 30 seconds, extension at 72°C for 30 seconds, and a final extension at 72°C for 10 minutes in the Veriti® 96-Well Thermal Cycler (Applied Biosystems, Foster City, USA). The PCR products were subjected to electrophoresis on a 1.5% agarose gel and stained with 0.5 mg/ml ethidium bromide. The amplified products were visualized using the Bio-Imaging System (DNR, Jerusalem, Israel). The PCR products were assessed by gel densitometry via ImageJ software [25] and quantified by Qubit™ dsDNA BR Assay Kit quantification (Thermo Fisher Scientific, Waltham, USA). PCR products were purified using the MinElute PCR Purification Kit (Qiagen, Hilden, Germany) when necessary.

### 2.6. Library Preparation for Illumina MiSeq Platform

The DNA libraries from the mock community of 20 bacterial DNA sequences were prepared with the TruSeq® DNA PCR-Free High-Throughput Library Prep Kit (Illumina, San Diego, USA) following the protocol for the purified amplicons. Each 16S rRNA gene region amplification product was labeled with a different index. Sequencing was conducted in the Illumina MiSeq System using the MiSeq Reagent Kit v2 (Illumina, San Diego, USA) (Table S2).

### 2.7. Library Preparation for Ion Torrent PGM Platform

The Ion Torrent PGM (Thermo Fisher Scientific, Waltham, USA) protocol for end repair, barcode and adapter attachment, clonal amplification, and sequencing was applied for the amplified fragments V1V2, V4V5, and V6V7V8 of the 16S rRNA gene (0–2.000 GE of *E*. *coli*, *S*. *enterica*, and *V*. *cholerae*) (Table S3). Samples containing human DNA were processed by the addition of 30 ng of previously fragmented human DNA of approximately 350 bp in length. The DNA was fragmented using the Bioruptor standard sonicator (Diagenode, Denville, USA) following the protocol recommended by the manufacturer. Fragments of 350 bases were selected using E-Gel SizeSelect on the E-Gel iBase Power System (Thermo Fisher Scientific, Waltham, USA). The libraries were constructed using the Ion Xpress Plus Fragment Library Kit (Life Technologies, Carlsbad, USA) followed by emulsion PCR on the Ion OneTouch 2 System using the Ion PGM Hi-Q Template Kit (Thermo Fisher Scientific, Waltham, USA). Enrichment was performed on the Ion OneTouch ES (Thermo Fisher Scientific, Waltham, USA), and sequencing was conducted on the Ion 318 Chip.

### 2.8. Sequencing Quality Control

The sequence reads were processed as follows: adaptors were removed, reads of less than 25 bases were discarded, and the remaining reads were trimmed at the 5' terminal (30 bases with Phred score lower than 15). Remaining reads were imported in FastQ format into the CLC Genomics Workbench software or into Geneious version 10.1.3. Reads shorter than 300 bp and 250 bp from the Ion Torrent PGM sequencing and Illumina MiSeq, respectively, were discarded.

### 2.9. Sequence Alignment

Reads from the mock community of 20 bacteria were mapped to their corresponding16S rRNA gene consensus sequences using CLC Genomics Workbench software with the following parameters: mismatch cost = 10, length fraction = 1.0, insertion cost = 3, deletion cost = 3, and similarity value (SV) = 0.95. To access primer specificity, reads from the simulated scenario with human DNA background were aligned to the 16S rRNA reference sequences selected from the Greengenes database (Supplementary Table 1) using Bowtie2 [26]. The “very-sensitive end-to-end” (-D 20 -R 3 -N 0 -L 20 -i S, 1, 0.50) mode was used (whole read length considered). The alignment files were analyzed either via CLC or Samtools [27] module idxstats.

### 2.10. Taxonomic Analysis

Taxonomic classifications were obtained using Kraken Metagenomics version 1.0, which is based on k-mers and achieves fast classification with reasonable accuracy [28] via the Galaxy web server (https://usegalaxy.org/). The bacteria Kraken database was selected, and other parameters were set to default. Graphs were generated using *R*.

## 3. Results

### 3.1. In Silico Validation of 16S rRNA Gene Hypervariable Regions

**Figure 1:**
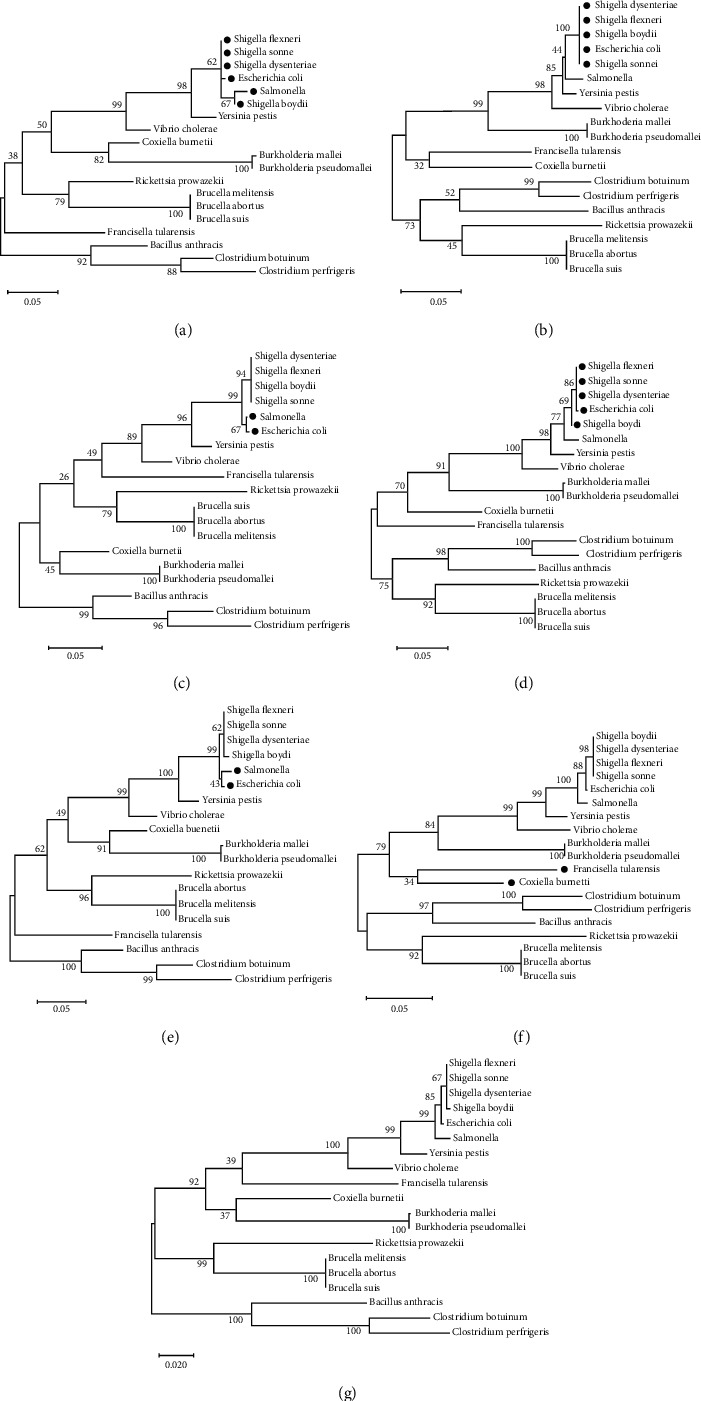
Phylogenetic tree of concatenated sequences of the 16S rRNA gene regions. The consensus sequences from the species described in Table 1 were aligned according to the variable region of 16S rRNA gene. Trees were generated using maximum likelihood, Kimura-2-parameter, gamma substitution, and bootstrap of 1000 replicates. (a) Variable regions (V1V2); (b) variable regions (V4V5); (c) variable regions (V6V7V8); (d) variable regions (V1V2 and V4V5); (e) variable regions (V1V2 and V6V7V8); (f) variable regions (V4V5 and V6V7V8); and (g) variable regions (V1V2, V4V5, and V6V7V8). The black dots indicate species that were not discriminated by specified regions to a monophyletic group.

Intraspecific and interspecific genetic similarities were evaluated based on the variation of the 16S rRNA gene nucleotide sequences of the selected 364 sequences representing 19 bacterial species and strains listed in the methods section that are potential agents for bioweapons. The similarities among species and strains were 74.23%–85.51%, and 94.48%–99.98%, respectively (Table 1). The resulting 16S rRNA consensus sequences from each bacterial species were aligned (Figure S1), and the nine hypervariable regions were determined. The conserved regions located in the three 16S rRNA gene variable sections (V1V2), (V4V5), and (V6V7V8) (Figure S2) were the targets for primer design (Table 2). To determine if the designed primer sets for the three regions would enable discrimination of the selected bacterial groups, a series of phylogenetic trees were generated using alignments of the 16S rRNA gene variable regions independently (V1V2), (V4V5), and (V6V7V8) and combined ((V1V2 and V4V5), (V1V2 and V6V7V8), and (V4V5 and V6V7V8)) (Figure 1). A combination of only two sets of variable regions failed to assign taxonomic groups correctly (Figures 1(a)–1(g)). The use of (V1V2) regions (Figure 1(a)), (V4V5) regions (Figure 1(b)), or (V6V7V8) regions (Figure 1(c)) failed to separate *Shigella* from *E*. *coli* and *Salmonella*. The addition of (V1V2) to (V4V5) (Figure 1(d)) and (V1V2) to (V6V7V8) (Figure 1(e)) did not resolve *E*. *coli* and *Salmonella* from the *Shigella* group. Furthermore, the combination of (V4V5) and (V6V7V8) (Figure 1(f)) was unable to separate *Burkholderia* from *F*. *tularensis* and *C*. *burnetii*. The *C*. *burnetii* and *F*. *tularensis* bootstrapping confidence value was 92% using (V1V2, V4V5, and V6V7V8) sequences and 34% using (V4V5 and V6V7V8) sequences only. Only a combination of the three primer pairs correctly assigned the monophyletic bacterial groups evaluated by the maximum likelihood trees (Figure 1(g)). All bacterial species were clustered within their own group, which corroborates the genetic similarity analysis described in Table 1. A combination of the three regions (V1V2), (V4V5), and (V6V7V8) (Figure 1(g)) of 16S rRNA led to high resolution separation of the taxonomic groups. These in silico results indicate that a combination of all these regions (with the primers described herein) supports the resolution of the selected group of bacteria that have potential to be used as bioweapons.

### 3.2. Efficacy of 16S rRNA Gene Primer Sets to Discriminate between Bacterial Species

**Figure 2:**
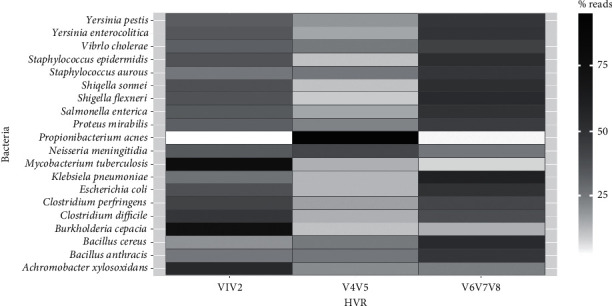
Percentage distribution of the reads mapped to 16S rRNA gene hypervariable regions (V1V2), (V4V5), and (V6V7V8) from a mock community containing 20 bacterial gDNA sequenced in Illumina MiSeq platform. Percentage of reads mapped to the respective bacterial 16S rRNA gene reference (similarity value = 0.95).

We evaluated the ability of degenerate primers to the 16S rRNA gene hypervariable regions (V1V2), (V4V5), and (V6V7V8) to discriminate between species in a simulated scenario of 2,000 GE of 20 bacteria. Sequence reads, generated on the MiSeq instrument (Table S3), were mapped to each of the three hypervariable regions at a rate of approximately 90% (Table S4). The number of mapped reads along the three hypervariable regions identified each bacterium in the mock community (Figure 2). The primers for the (V4V5) hypervariable region showed less affinity for the bacterial species used in the experiment, except for *P*. *acnes* and *B*. *cepacia*. The primers for (V1V2) and (V6V7V8) hypervariable regions distinguished between most bacteria of the same genus, e.g., *Y*. *enterocolitica* and *Y*. *pestis* or *S*. *flexneri* and *S*. *sonnei* more efficiently than other combinations. The degenerate primers had preferential affinity for some species and hypervariable regions. Nevertheless, the pattern of amplification was similar among the three hypervariable regions when comparing one species to another, as for *C*. *perfringens* and C. *difficile* or *B*. *anthracis* and *B*. *cereus*. Although the V4-5 primers showed a bias to *P*. *acnes*, the other primer sets did not. Then, the use of the three set together enhances the ability to discriminate our target bacteria. The average confidence interval (95%) errors for the three hypervariable regions (V1V2, V4V5, and V6V7V8) were 0.070 ± 0.002, 0.135 ± 0.008, and 0.051 ± 0.001, respectively. These observations may reflect the different efficiencies of the primer pairs to target the different 16S rRNA regions among the bacterial groups; therefore, they should be used in combination for bacterial detection. As the 16S rRNA gene is universally present in bacteria, one can also evaluate the diversity of the bacterial community in any given sample. We evaluated the discriminatory power of these primers using an environmental sample. Using Kraken for taxonomic classification of the reads, a broad diversity of bacteria was identified. In addition, the different affinities of the primer pairs for the 16S rRNA gene hypervariable regions were reflected in the bacterial groups present in this environmental sample (Figure S3).

### 3.3. Detection Level of the 16S rRNA Gene Hypervariable Regions Primer Sets

**Figure 3:**
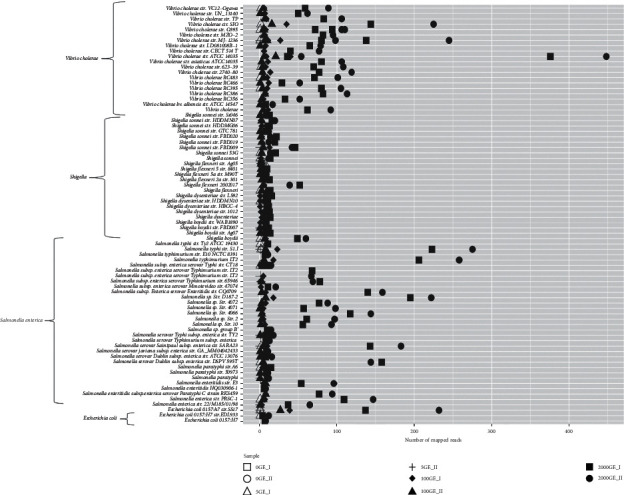
Top reads of the simulated bacterial DNA mock community (*S*. *enterica*, *V*. *cholerae*, and *E*. *coli)* in human DNA background sequenced in Ion Torrent PGM platform mapped according 364 target sequences. Shigella mapping is also included. Different GE concentrations are in duplicate. OGE_I and II; 5 GE_I and II; 100 GE_I and II; 2,000 GE_I and II.

**Figure 4:**
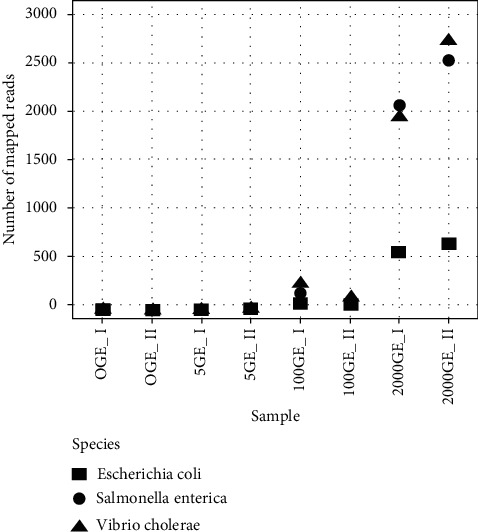
Number of mapped reads for target bacterial species in a human DNA background sample sequenced in Ion Torrent PGM. Alignment was produced using the 16S rRNA consensus gene of the three species *Escherichia coli*, *Salmonella enterica*, and *Vibrio cholera* using Bowtie2. Different GE concentrations are in duplicate. 0 GE_I and II; 5 GE_I and II; 100 GE_I and II; 2,000 GE_I and II.

To assess the applicability of the system, we evaluated the ability of the primer sets to detect a diverse group of bacteria in a human DNA background sample that simulated infected subjects. We added three distinct bacterial DNA species (*E*. *coli*, *V*. *cholera*, and *S*. *enterica*) to the mock scenario at increasing GE amounts (5, 100, and 2,000) of gDNA to a human DNA background sample (Table S3). The reads obtained by massively parallel sequencing were mapped to a complex database of 16S rRNA genes comprising the 364 reference sequences (Table S1) using Bowtie2. The alignment of the reads in the absence of bacterial gDNA (0 GE) or at low genome equivalent amount (5 GE) was insufficient to map to the 16S rRNA gene references. In these conditions, the majority of the sequencing reads came from a human source, as expected, and only 10%–12% of the generated sequences were mapped to the 16S rRNA gene (Table S5). From these, we detected *S*. *enterica*, *E*. *coli*, and *V*. *cholerae* as the top mapped reads for the mock community at 100 GE of simulated bacterial DNA (up to 50); and the target bacteria were mapped at a 10-fold–100-fold increased frequency at 2,000 GE (Figure 3). Because *E*. *coli* and *Shigella* are closely related genera, the reads that mapped to *Shigella* (less than 50 reads at 2,000 GE) were considered to represent the false-positive threshold, as *Shigella* DNA was not present in the mock community. Next, we evaluated the sensitivity of the primer sets to detect the three species of interest by aligning the reads only to the 16SrRNA gene consensus sequences for the selected species, calculated as explained previously in the Methods section (Figure 4). Reads obtained from the Ion PGM sequencing platform DNA analysis of the bacterial DNA mock community (*S*. *enterica*, *V*. *cholerae*, and *E*. *coli)* were aligned to the 16S rRNA consensus sequences using Bowtie2. The detection of *E*. *coli*, *S*. *enterica*, and *V*. *cholera* became evident at 2,000 GE.

Furthermore, we investigated if the sensitivity of the primer sets was evident for a more complex sample. For that, we analyzed two mock scenarios of the three species of interest (*E*. *coli*, *S*. *enterica*, and *V*. *cholera)* in different GE concentrations (0 and 2,000) in a DNA background extracted from an environmental sample (Table S3).

Reads obtained using MiSeq platform sequencing were mapped to the 16S rRNA gene consensus for the three species (*E*. *coli*, *S*. *enterica*, and *V*. *cholera)* and Bowtie2. The detection of the three species was evident at 2,000 GE, attesting to the validity of our protocol (Figure S4).

## 4. Discussion

Bacterial detection in clinical or environmental samples by shotgun MPS is limited when the target bacterial DNA is present in low copy number. Partial 16S rRNA gene sequences are used directly in microbiome analysis [29]. Because different regions of the 16S rRNA gene have different divergences, the choice of target partial sequence region can substantially affect the analysis results [30–33]. Thus, it is important and useful to determine how reliably a partial 16S rRNA gene region can support the characterization of bacterial groups compared with near full-length 16S rRNA genes. Recently, it has been proposed that full-length 16S intragenomic copy variants have the potential to provide taxonomic resolution for microbiome analysis to the species and strain level [17]. In this study, we compared all partial sequence regions spanning seven hypervariable regions (V1, V2, V4, V5, V6, V7, and V8) and selected in silico, the best combinations from 364 sequences of 19 bacterial species and strains of microbial forensic interest. Primers were designed based on conserved regions and tested against mock samples composed of 20 bacterial strains. Our study targeted the 16S rRNA gene and, therefore, did not differentiate pathogenic from nonpathogenic *B*. *anthracis* strains, which is conferred by two virulence plasmids (pXO1 and pXO2). Samples were amplified and subsequently sequenced with two MPS platforms (Ion Torrent PGM and MiSeq). Nevertheless, the resolution and sensitivity, expressed as minimum bacterial GE detection, were assessed using a novel 16S rRNA gene primer set that covered the 16S rRNA gene (V1V2), (V4V5), and (V6V7V8) regions followed by MPS. We evaluated the resolution power of these primers for an environmental sample using Kraken for taxonomic classification of the reads, which is based on *k*-mers and achieves fast classification with reasonable accuracy. Our data show that the sensitivity of both platforms was 2,000 GE. Read mapping using a complex database of 364 curated 16S rRNA gene sequences successfully detected the target bacteria species *Salmonella enterica*, *Vibrio cholerae*, and *E*. *coli* as the top mapped reads at 2,000 GE. Our approach allows for better assessment of background noise, which is important when an accurate analysis of such samples is needed [11, 12, 34–38]. Our results show that the sensitivity of detection gained from a more comprehensive coverage of the 16S rRNA gene, using different GE of *E*. *coli*, *V*. *cholerae*, and *S*. *enterica* DNA, is proportional to the input of target DNA. Therefore, one can detect or monitor as low as 2,000 GE for the species tested herein on samples of unknown origin; however, results are improved at 2,000 GE target DNA concentration. Previous work, using the V2, V3, and V6 regions together, suggested that a combination of two or more variable regions in a multiplex assay would have better resolution power at the genus level [15]. Another study evaluating microbiome analysis using multiple hypervariable regions (V2, V4, and V6-7 but not V9) gave satisfactory results [39]. Data from an in-house protocol for the hypervariable V4 primer region provided the bacterial diversity of different biological fluids: *Streptococcus*, *Veillonella*, and *Haemophilus* in saliva; *Lactobacillus*, *Gardnerella*, and *Finegoldia* in menstrual secretion; and *Lactobacillus*, *Prevotella*, and *Gardnerella* in vaginal secretion [40]. The in-house primers targeting the V4-V5 regions of the 16S rRNA gene allowed the identification of *Lactobacillus iners*, present in the vaginal fluid but not in the saliva samples [41]. Therefore, microbial composition data may differ depending on the degenerate 16S rRNA gene primers and sequencing platform used [14], which motivated our work to design specific degenerate primers to detect bacteria on different MPS platforms for potential use in bioweapon detection or clinical diagnostics. A previous study using phylogenetically discriminating SNPs and mock scenarios of 1,000,000, 10,000, or 100 GE of *B*. *anthracis*, *Y*. *pestis*, *F*. *tularensis*, and *B*. *pseudomallei* mixed with 3,000 GE of human gDNA and 10,000 GE of *Thermotoga maritima* [42] suggested that MPS may have limitations in species identification when applied to complex environmental or clinical samples. However, their panel focused on targeting amplicons of specific genes or SNPs instead of, or in addition to, targeting those of specific organisms. The authors reported detecting only *B*. *pseudomallei* from a sample with 100 GE of the bacterium, and no other sequences were mapped with fewer GE. Furthermore, in their work, less stringent parameters such as length fraction = 0.50 and similarity = 0.80 were used. A broader range of bacteria may be detected using lower similarity/identity values than the ones applied herein. In addition, the data obtained from these primers and MPS suggest they may be used as classifiers [43] to reveal the background bacteria of a sample, but the application may be limited depending on the data in the 16S rRNA gene database. Variation in the number of species reported by different methods could be attributed to both the differences in the taxonomy assignment strategy and the reference databases used by the methods [13]. It is likely that our 16S rRNA gene-targeted primers, in combination with MPS, can be used to detect bacteria from complex samples, as evidenced by testing with an environmental background. We also tested the taxonomic classification ability of the primer set using Kraken for an environmental sample showing a broad range of bacterial diversity. The performance was superior to that of using any single subregion of the 16S rRNA gene. These selected primer pairs may function as universal markers that can be used to discriminate between groups of bacteria in an unknown sample with a single test. Finally, the short reads (≤250 bp) obtained by amplification of the targeted 16S rRNA gene hypervariable regions from these bacterial references showed that the sensitivity was independent of the background scenario. Our approach may provide a way to relatively quantify species of bacteria from different groups by comparisons based on read mapping.

## 5. Conclusions

The ability to detect pathogenic microorganisms has strategic importance for both health and security perspectives. This work provides a robust protocol to detect and/or monitor, to as low as 2,000 GE, a selected set of pathogenic bacterial species associated with clinical, bioweapon, and hazardous samples.

## Figures and Tables

**Table 1:** Intraspecific and interspecific genetic similarity values of 16S rRNA gene.

Sequences extracted from the Greengenes database										
#	Bacteria	Number of sequences		Average similarity ± standard deviation (%)						Consensus sequence (bp)
		Total	Filtered	Intraspecific			Interspecific			
1	*B*. *anthracis*	234	17	99.56	±	0.6	75.81	±	2.58	1558
2	*B*. *melitensis*	90	12	99.60	±	0.5	82.55	±	7.81	1486
3	*B*. *suis*	16	4	99.50	±	0.7	80.84	±	7.18	1500
4	*B*. *abortus*	20	7	99.96	±	0.04	82.11	±	8.62	1496
5	*B*. *mallei*	67	14	99.98	±	0.04	79.39	±	5.8	1539
6	*B*. *pseudomallei*	132	31	99.69	±	0.51	79.52	±	5.77	1544
7	*C*. *botulinum*	224	28	94.48	±	4.84	74.23	±	5.07	1534
8	*C*. *perfringens*	480	57	99.72	±	0.2	74.97	±	4.57	1357
9	*C*. *burnetii*	19	11	99.72	±	0.2	80.89	±	2.82	1546
10	*E*. *coli*	9830	7	99.65	±	0.37	85.24	±	10.83	1543
11	*F*. *tularensis*	49	25	99.76	±	0.17	78.64	±	2.98	1536
12	*R*. *prowazekii*	7	7	99.25	±	1.22	75.64	±	3.39	1501
13	*S*. *enterica*	1025	47	99.43	±	0.43	85.16	±	10.29	1593
14	*S*. *boydii*	59	12	99.66	±	0.22	85.51	±	10.69	1545
15	*S*. *dysenteriae*	26	11	99.55	±	0.28	85.45	±	10.81	1551
16	*S*. *flexneri*	65	12	99.33	±	0.74	85.45	±	10.81	1552
17	*S*. *sonnei*	32	11	99.82	±	0.15	85.45	±	10.81	1547
18	*V*. *cholerae*	105	24	99.87	±	0.11	83.63	±	7.33	1549
19	*Y*. *pestis*	152	27	99.90	±	0.07	84.33	±	9.65	1592
	Total of sequences	12632	364							

**Table 2:** Degenerate 16S rRNA primers for PCR assays.

16S rDNA regions	Degenerate primer sequence (5′-3′)		Tm (^o^C)	Amplicon length (bases)
(V1V2)	F:	TWACACATGCAAGTCGARCG	56–59	339
	R:	CAAWATTCCCCACTGCTGCC	58–59	
(V4V5)	F:	CAGCCGCGGTAATACGDAGG	60–63	380
	R:	TGCGRCCGTACTCCCCAGGC	60–63	
(V6V7V8)	F:	CGA WGCAACGCGAARAACCT	60–61	362
	R:	CGAGTTGCAGACTVCAATCCG	59–62	

Degenerate bases (W = A/T, R = A/G, and D = A/G/T)

## Data Availability

The data used to support this study are made available from the corresponding author upon request or can be found on ftp://146.164.75.45 (user: ftp; password: ftp).

## Additional Points

We described a set of 16S rRNA gene primers obtained via alignment of 364 sequences representing 19 bacterial species and strains relevant to medical and forensics applications. In silico results support the resolution of a selected group of bacteria. Interspecies and intraspecies comparisons showed 74.23%–85.51% and 94.48%–99.98% sequencing variation among species and strains, respectively. This protocol can detect or monitor 2,000 genome equivalents of bacterial species associated with clinical, bioweapon, and biohazards samples. Potentially, we can distinguish natural outbreaks of pathogenic microorganisms from those occurring by intentional release.

## Supplementary Materials

Supplementary Materials Table S1: 16S rRNA gene sequences used as a source for the selection of variable regions from each of the 19 selected strains of bacterial species. Table S2: bacterial DNA source for the simulated metagenomic community. Figure S1: alignment of the consensus sequences of the 16S rRNA gene. The conserved regions are indicated by a color scale, from green (more conserved) to yellow (less conserved), and the most varied or gaps are represented in white. The blue rectangles correspond to the identified hypervariable regions of the alignment. Figure S2: the 16S rRNA gene locus and location of hypervariable regions and primers developed in this study. Blue rectangles represent the hypervariable regions V1–V9; the forward primers are indicated in dark green and the reverse primers in light green. Table S3: source of samples, target region of 16S rRNA gene primers, and sequencing platform used in the study experiments. Table S4: percentage mapped and unmapped reads of the bacterial mock community sequenced in Illumina MiSeq platform. Reads discriminated using the primer regions, tested individually in two replicates, and denominated by the suffixes I and II. Figure S3: taxonomic bacterial classification of an environmental sample sequenced in Illumina MiSeq platform. Distribution determined using combinations of primer sets targeting 16S rRNA gene hypervariable regions (V1-V2-V4-V5-V6-V7-V8). Classifications were obtained using Kraken. Table S5: sensitivity of the primer sets to detect a diverse group of bacteria in a human DNA background to simulate infected subjects. Figure S4: number of reads mapped to the consensus 16S rRNA in an environmental sample without addition of mock bacterial community (0 GE) and with simulated bacterial scenarios sequenced in Illumina MiSeq platform. The most accurate combinations of primer sets targeting 16S rRNA gene hypervariable regions (V1-V2-V4-V5-V6-V7-V8) are revealed by mapping reads to consensus 16S rRNA using only Bowtie2. The 2,000 GE mock community (*S*. *enterica*, *E*. *coli*, and *V*. *cholerae*) was added to the same environmental sample used for 0 GE.
