# Supplementary material for: Evaluation of 16S rRNA Hypervariable Regions for Bioweapon Species Detection by Massively Parallel Sequencing
Source: Int J Microbiol. 2020 Sep 26;2020:8865520. doi: 10.1155/2020/8865520 (PMC7533751; doi:10.1155/2020/8865520)
Supplement: Supplementary Materials — Table S1: 16S rRNA gene sequences used as a source for the selection of variable regions from each of the 19 selected strains of bacterial species. Table S2: bacterial DNA source for the simulated metagenomic community. Figure S1: alignment of the consensus sequences of the 16S rRNA gene. The conserved regions are indicated by a color scale, from green (more conserved) to yellow (less conserved), and the most varied or gaps are represented in white. The blue rectangles correspond to the identified hypervariable regions of the alignment. Figure S2: the 16S rRNA gene locus and location of hypervariable regions and primers developed in this study. Blue rectangles represent the hypervariable regions V1–V9; the forward primers are indicated in dark green and the reverse primers in light green. Table S3: source of samples, target region of 16S rRNA gene primers, and sequencing platform used in the study experiments. Table S4: percentage mapped and unmapped reads of the bacterial mock community sequenced in Illumina MiSeq platform. Reads discriminated using the primer regions, tested individually in two replicates, and denominated by the suffixes I and II. Figure S3: taxonomic bacterial classification of an environmental sample sequenced in Illumina MiSeq platform. Distribution determined using combinations of primer sets targeting 16S rRNA gene hypervariable regions (V1-V2-V4-V5-V6-V7-V8). Classifications were obtained using Kraken. Table S5: sensitivity of the primer sets to detect a diverse group of bacteria in a human DNA background to simulate infected subjects. Figure S4: number of reads mapped to the consensus 16S rRNA in an environmental sample without addition of mock bacterial community (0 GE) and with simulated bacterial scenarios sequenced in Illumina MiSeq platform. The most accurate combinations of primer sets targeting 16S rRNA gene hypervariable regions (V1-V2-V4-V5-V6-V7-V8) are revealed by mapping reads to consensus 16S rRNA using only Bowtie2. The 2,000 [file 8865520.f1.zip › 8865520.f1/Table S1.docx]

Table S1- 16S rRNA gene sequences used as source for the selection of variable regions from each of the 19 selected strain of bacterial species.

| **Name** | **Genbank ID** |
| --- | --- |
| **Bacillus anthracis** |  |
| **Bacillus anthracis str. B1** | DQ105975.1 |
| **Bacillus anthracis str. A0442** | NZ ABKG01000043.1 |
| **Bacillus anthracis str. A0488** | ABJC01000055.1 |
| **Bacillus anthracis str. Tsiankovskii-I** | NZ ABDN01000028.1 |
| **Bacillus anthracis str. A0465** | ABLH01000020.1 |
| **Bacillus anthracis str. Sterne** | NC 005945.1 |
| **Bacillus anthracis str. Sterne** | AE017225.1 |
| **Bacillus anthracis str. A0193** | ABKF01000014.1 |
| **Bacillus anthracis str. Ames** | AF155950.1 |
| **Bacillus anthracis str. Ames** | AF155951.1 |
| **Bacillus anthracis str. Sterne** | AF176321.1 |
| **Bacillus anthracis str. A0442** | ABKG01000011.1 |
| **Bacillus anthracis str. A0465** | NZ ABLH01000024.1 |
| **Bacillus anthracis str. A0389** | ABLB01000010.1 |
| **Bacillus anthracis 'Ames Ancestor' str. Ames Ancestor A2084** | NC 007530.2 |
| **Bacillus anthracis str. Ames** | NC 003997.3 |
| **Bacillus anthracis** | AY043083.1 |
| **Burkholderia mallei str. 10229** | NC_008836.1 |
| **Brucella abortus** |  |
| **Brucella abortus biovar 1 str. 9-941** | NC 006933.1 |
| **Brucella abortus biovar 1 str. 9-941** | NC 006932.1 |
| **Brucella abortus str. CNM 20040339** | EF192471.1 |
| **Brucella abortus str. S19** | NC 010742.1 |
| **Brucella abortus clone 3-1** | EU816701.1 |
| **Brucella abortus bv. 2 str. 86/8/59** | NZ ACBJ01000075.1 |
| **Brucella abortus (T) type strain: NCTC 10093** | UFTU01000001 |
| **Brucella melitensis** |  |
| **Brucella melitensis 2528** | L26166.1 |
| **Brucella melitensis biovar Abortus str. 11-19** | X13695.1 |
| **Brucella melitensis biovar Abortus str. 2000031282** | AY513567.1 |
| **Brucella melitensis str. 16M** | AE009462.1 |
| **Brucella melitensis biovar Abortus str. 2308** | AM040265.1 |
| **Brucella melitensis biovar Suis str. NCTC 10316** | AM158980.1 |
| **Brucella melitensis biovar Abortus str. NCTC 10093** | AM158979.1 |
| **Brucella melitensis str. AUH2** | EF187230.1 |
| **Brucella melitensis Kars1** | AY922323.1 |
| **Brucella melitensis str. ATCC 23457** | CP001489.1 |
| **Brucella melitensis bv. 1 str. 16M** | AE008918.1 |
| **Brucella melitensis str. ATCC 23457** | CP001488.1 |
| **Brucella suis** |  |
| **Brucella suis** | L26169.1 |
| **Brucella suis str. 1330** | NC 004310.3 |
| **Brucella suis str. ATCC 23445** | NC 010167.1 |
| **Brucella suis bv. 5 str. 513** | NZ ACBK01000034.1 |
| **Burkholderia mallei** | AF110187.1 |
| **Burkholderia mallei str. ATCC 23344** | AF110188.1 |
| **Burkholderia mallei str. 2002721278** | AY305754.1 |
| **Burkholderia mallei str. ATCC 23344** | NC 006348.1 |
| **Burkholderia mallei str. ATCC 23344** | NC 006349.2 |
| **Burkholderia mallei str. SAVP1** | NZ AAHQ01000001.1 |
| **Burkholderia mallei str. 10229** | NZ AAHM01000002.1 |
| **Burkholderia mallei str. SAVP1** | NZ AAHQ02000001.1 |
| **Burkholderia mallei str. SAVP1** | CP000525.1 |
| **Burkholderia mallei str. SAVP1** | NC 008784.1 |
| **Burkholderia mallei str. NCTC 10229** | NC 008835.1 |
| **Burkholderia mallei str. NCTC 10229** | NC 008835.1 |
| **Burkholderia mallei str. NCTC 10247** | CP000547.1 |
| **Burkholderia mallei str. PRL-20** | NZ AAZP01000007.1 |
| **Burkholderia pseudomallei** |  |
| **Burkholderia pseudomallei** | AJ131790.1 |
| **Burkholderia pseudomallei str. 1026b** | U91839.1 |
| **Burkholderia pseudomallei K96243** | AF093055.1 |
| **Burkholderia pseudomallei L2** | AF093054.1 |
| **Burkholderia pseudomallei H2** | AF093053.1 |
| **Burkholderia pseudomallei V685** | AF093057.1 |
| **Burkholderia pseudomallei V824** | AF093059.1 |
| **Burkholderia pseudomallei V688** | AF093058.1 |
| **Burkholderia pseudomallei H1** | AF093047.1 |
| **Burkholderia pseudomallei V830** | AF093060.1 |
| **Burkholderia pseudomallei** | AY198339.1 |
| **Burkholderia pseudomallei str. 2002721184** | AY305776.1 |
| **Burkholderia pseudomallei str. K96243** | AY305764.1 |
| **Burkholderia pseudomallei str. C7532** | AJ616911.2 |
| **Burkholderia pseudomallei str. C6756** | AJ616912.2 |
| **Burkholderia pseudomallei (Ara+ biotype) V681** | AF093049.1 |
| **Burkholderia pseudomallei str. K96243** | BX571965.1 |
| **Burkholderia pseudomallei str. 1710b** | NC 007434.1 |
| **Burkholderia pseudomallei str. ATCC 23343** | DQ108392.1 |
| **Burkholderia pseudomallei str. 305** | NZ AAYX01000015.1 |
| **Burkholderia pseudomallei str. 1106a** | NC 009076.1 |
| **Burkholderia pseudomallei str. 1106a** | NC 009078.1 |
| **Burkholderia pseudomallei str. 668** | CP000570.1 |
| **Burkholderia pseudomallei str. 305** | AAYX01000015.1 |
| **Burkholderia pseudomallei str. BX 571966** | EU024169.1 |
| **Burkholderia pseudomallei str. 112** | NZ ABBP01001245.1 |
| **Burkholderia pseudomallei NCTC 13177 str. NCTC13177** | NZ ABBQ01001003.1 |
| **Burkholderia pseudomallei str. BCC215** | NZ ABBR01001005.1 |
| **Burkholderia pseudomallei str. B7210** | NZ ABBN01000253.1 |
| **Burkholderia pseudomallei str. MSHR346** | CP001408.1 |
| **Burkholderia pseudomallei str. CEMM1333** | GU123622.1 |
| **Clostridium botulinum** |  |
| **Clostridium botulinum str. DSM 1734** | X73442.1 |
| **Clostridium botulinum** | L37593.1 |
| **Clostridium botulinum** | L37587.1 |
| **Clostridium botulinum str. KYTO-F** | X73844.1 |
| **Clostridium botulinum str. LP1284** | AF105402.1 |
| **Clostridium botulinum str. NCTC7272** | X68185.1 |
| **Clostridium botulinum B str. BP2** | L37588.1 |
| **Clostridium botulinum str. A2** | L37586.1 |
| **Clostridium botulinum type A** | L37585.1 |
| **Clostridium botulinum str. Langeland NCTC10281** | X68172.1 |
| **Clostridium botulinum B str. B3** | L37589.1 |
| **Clostridium botulinum str. NCTC7273** | X68186.1 |
| **Clostridium botulinum str. Iwanai** | X68170.1 |
| **Clostridium botulinum** | L37592.1 |
| **Clostridium botulinum str. Eklund 202F ATCC23387** | X68171.1 |
| **Clostridium botulinum str. Eklund 17B ATCC25765** | X68173.1 |
| **Clostridium botulinum str. B155** | EF051574.1 |
| **Clostridium botulinum str. B163** | EF051572.1 |
| **Clostridium botulinum str. E213** | EF030540.1 |
| **Clostridium botulinum str. B258** | EF051573.1 |
| **Clostridium botulinum str. E185** | EF030539.1 |
| **Clostridium botulinum C str. Eklund** | NZ ABDQ01000030.1 |
| **Clostridium botulinum B1 str. Okra** | NC 010516.1 |
| **Clostridium botulinum C str. Eklund** | NZ ABDQ01000010.1 |
| **Clostridium botulinum E3 str. Alaska E43** | NC 010723.1 |
| **Clostridium botulinum str. BG-C109** | FJ384382.1 |
| **Clostridium botulinum A2 str. Kyoto** | CP001581.1 |
| **Clostridium botulinum str. ELTDK 103 ATCC 25763** | NR 029157.1 |
| **Clostridium perfringens** |  |
| **Clostridium sp. str. AB&J** | AF390549.1 |
| **swine intestine clone p-4636-2Wa2** | AF371845.1 |
| **Clostridium perfringens str. 13** | AB045282.1 |
| **feces clone 1E2** | AY581817.1 |
| **dog duodenum clone D1-03** | DQ113738.1 |
| **dog colon clone C5-04** | DQ113680.1 |
| **dog ileum clone I8-10** | DQ113759.1 |
| **Clostridium perfringens str. B22** | DQ196140.1 |
| **Fermentative hydrogen production anaerobic sludge foodwaste Clostridium clone N31** | DQ232861.1 |
| **Clostridium perfringens U523B2** | DQ298091.1 |
| **human fecal clone RL245 aai81f07** | DQ795743.1 |
| **Clostridium perfringens JS7** | AM889032.1 |
| **Clostridium perfringens CPE str. F4969** | NZ ABDX01000014.1 |
| **Clostridium perfringens E str. JGS1987** | ABDW01000023.1 |
| **Clostridium perfringens str. NCTC 8239** | ABDY01000009.1 |
| **Clostridium perfringens B str. ATCC 3626** | NZ ABDV01000036.1 |
| **polar bear feces clone PB2 aai23b08** | EU460596.1 |
| **hedgehog feces clone HH b03** | EU467033.1 |
| **hedgehog feces clone HH c07 1** | EU467060.1 |
| **polar bear feces clone PB2 aai21e01** | EU460481.1 |
| **cheetah feces clone CE3 aai08f09** | EU459431.1 |
| **flying fox feces clone FF a07 1** | EU469743.1 |
| **hedgehog feces clone HH f09** | EU467039.1 |
| **bush dog feces clone bdog3 aad67a05** | EU468102.1 |
| **cheetah feces clone CE2 c04 2** | EU468017.1 |
| **hedgehog feces clone HH g08** | EU467040.1 |
| **cheetah feces clone CE2 d09 3** | EU468078.1 |
| **polar bear feces clone PB2 aai21b03** | EU460455.1 |
| **bush dog feces clone bdog1 g02 2** | EU467735.1 |
| **hedgehog feces clone HH c10** | EU467036.1 |
| **bush dog feces clone bdog1 a08 1** | EU467737.1 |
| **Hedgehog feces clone HH aai35d06** | EU775668.1 |
| **Clostridium perfringens str. DJF B043** | EU728702.1 |
| **North American Black Bear feces clone BB1 e11** | EU772579.1 |
| **Lion feces clone LI3 aah77h05** | EU776582.1 |
| **Cheetah feces clone CE2 f02** | EU773824.1 |
| **Bush dog feces clone bdog1 aai79a01** | EU772880.1 |
| **Bush dog feces clone bdog1 aai80b01** | EU772738.1 |
| **Lion feces clone LI3 aah77h12** | EU776537.1 |
| **blaTEM genes polar bear feces clone PBF d5** | FJ375868.1 |
| **Cheetah feces clone CE2 c01** | EU773814.1 |
| **Clostridium perfringens str. ES MS26c** | EU888517.1 |
| **blaTEM genes polar bear feces clone PBF d14** | FJ375877.1 |
| **Cheetah feces clone CE2 h08 1** | EU773975.1 |
| **Clostridium perfringens str. ES MS22c** | EU888515.1 |
| **blaTEM genes polar bear feces clone PBF b43** | FJ375812.1 |
| **Lion feces clone LI3 aah77g05** | EU776618.1 |
| **feces adult twins and mothers TS148 clone TS148 a01e12** | FJ362987.1 |
| **feces clone CL F 396** | FJ978702.1 |
| **Clostridium perfringens str. JPL 18** | FJ957872.1 |
| **feces clone CL F 096** | FJ978550.1 |
| **Human gut microbiome adopts alternative state bowel transplantation  ileum intestinal transplant clone SHZN601** | GQ156514.1 |
| **Clostridium perfringens str. MOTB5** | FJ215325.1 |
| **Clostridium sp. AG07-9** | FM865910.1 |
| **Clostridium perfringens str. JI12** | FJ215343.1 |
| **Clostridium perfringens str. JI1** | FJ215347.1 |
| **Clostridium perfringens str. SG7** | FJ215324.1 |
| **Coxiella burnetii** |  |
| **Coxiella burnetii** | M21291.1 |
| **Coxiella burnetii str. RSA 493** | NC 002971.3 |
| **Coxiella burnetii str. VR145** | AY342037.1 |
| **Coxiella burnetii str. ATCC VR-616 Nine Mile** | Y11502.1 |
| **Coxiella burnetii str. S1** | Y11500.1 |
| **Coxiella burnetii str. RSA 493** | AE016828.2 |
| **Coxiella burnetii str. Dugway 5J108-111** | NC 009727.1 |
| **Coxiella burnetii str. RSA 331** | NC 010117.1 |
| **Coxiella burnetii str. CbuG Q212** | NC 011527.1 |
| **Coxiella burnetii str. CbuK Q154** | NC 011528.1 |
| **Coxiella burnetii str. Ammassalik** | FJ787329.1 |
| **Escherichia coli** |  |
| **Escherichia coli O157:H7 str. Sakai** | NC_002695.2 |
| **Escherichia coli O157:H7 str. Sakai RIMD 0509952** | BA000007.3 |
| **Escherichia coli O157:H7** | AE005174.2 |
| **Escherichia coli O157:H7 str. SS17** | NZ_CP008805.1 |
| **Escherichia coli O157:H7 str. EDL933** | NZ_CP008957.1 |
| **Escherichia coli str. O157:H7** | AY513502.1 |
| **Escherichia coli O157:H7 str. EC4501** | NZ ABHT01000152.1 |
| **Francisella tularensis** |  |
| **Francisella tularensis** | L26084.1 |
| **Francisella tularensis** | L26086.1 |
| **Francisella tularensis str. FTMC1** | AF143093.1 |
| **Francisella tularensis str. SCHU FSC 043** | Z21932.1 |
| **Francisella tularensis str. lvs ATCC 6223 FSC 155** | Z21931.1 |
| **Francisella tularensis str. 3523** | AY243028.1 |
| **Francisella tularensis subsp. mediasiatica str. FSC 147** | AJ698863.1 |
| **Francisella tularensis subsp. holarctica str. FSC 090** | AJ698864.1 |
| **Francisella tularensis subsp. holarctica str. FSC 025** | AY968229.1 |
| **Francisella tularensis subsp. mediasiatica str. FSC 148** | AY968235.1 |
| **Francisella tularensis subsp. holarctica str. FSC 257** | AY968231.1 |
| **Francisella tularensis subsp. mediasiatica str. FSC 122** | AY968233.1 |
| **Francisella tularensis subsp. holarctica str. FSC 022** | AY968228.1 |
| **Francisella tularensis subsp. holarctica str. FSC 017** | AY968227.1 |
| **Francisella tularensis subsp. mediasiatica str. FSC 149** | AY968236.1 |
| **Francisella tularensis subsp. holarctica str. UT01-1901** | AY968232.1 |
| **Francisella tularensis subsp. mediasiatica str. FSC 147** | AY968234.1 |
| **Francisella tularensis str. FSC 156 2766 fx1** | AY968238.1 |
| **Francisella tularensis subsp. novicida str. CIP 56.12** | AY928396.1 |
| **Francisella tularensis subsp. holarctica str. LVS** | NC 007880.1 |
| **Francisella tularensis subsp. novicida str. U112 k** | CP000439.1 |
| **Francisella tularensis subsp. holarctica str. FTA** | NC 009749.1 |
| **Francisella tularensis subsp. holarctica str. OSU18** | NC 008369.1 |
| **Francisella tularensis FSC147 subsp. mediasiatica str. FSC147** | NC 010677.1 |
| **Francisella tularensis subsp. novicida str. FTE M3** | ABSS01000002.1 |
| **Rickettsia prowazekii** |  |
| **Rickettsia prowazekii** | M21789.1 |
| **Rickettsia prowazekii str. Madrid/** | AJ235272.1 |
| **Rickettsia prowazekii Madrid E** | GCF_000195735.1 |
| **Rickettsia prowazekii SDT2S4** | JQ045807.1 |
| **Rickettsia prowazekii str. Chernikova** | NC_017049.1 |
| **Rickettsia prowazekii str. Dachau** | CP003394.1 |
| **Rickettsia prowazekii str. RpGvF24** | NC_017057.1 |
| **Salmonella enterica** |  |
| **Salmonella enterica arizonae subsp. IIIa str. ATCC 13314T** | AF008580.1 |
| **Salmonella enterica subsp. arizonae str. DSM 9386** | EU014683.1 |
| **Pantoea agglomerans str. A11** | AF130890.2 |
| **Salmonella subsp. enterica str. serovar Shomron** | X80678.1 |
| **Salmonella enterica subsp. arizonae serovar 62:z4z23 str. RSK2980** | NC 010067.1 |
| **Salmonella enterica subsp. indica str. DSM 14848** | EU014680.1 |
| **Salmonella enterica subsp. houtenae str. DSM 9221** | EU014684.1 |
| **Salmonella paratyphi str. 50973** | DQ683179.1 |
| **Salmonella paratyphi** | DQ344536.1 |
| **Salmonella enterica subsp. salamae str. GTC 1731** | AB273734.1 |
| **Salmonella enterica subsp. salamae str. DSM 9220** | EU014685.1 |
| **Salmonella typhimurium str. E10 NCTC 8391** | Z49264.1 |
| **Salmonella typhi str. Ty2 ATCC 19430** | Z47544.1 |
| **Salmonella subsp. enterica serovar Montevideo str. 47074 subsp.** | AF227867.1 |
| **Salmonella paratyphi str. A6** | EU118081.1 |
| **Salmonella typhimurium LT2 str. LT2 SGSC 1412 ATCC** | AE008881.1 |
| **Salmonella serovar Typhi subsp. enterica str. Ty2** | NC 004631.1 |
| **Salmonella subsp. enterica serovar Typhi str. CT18 subsp.** | AL627278.1 |
| **Salmonella subsp. enterica serovar Typhi str. CT18 subsp.** | AL627266.1 |
| **Salmonella serovar Typhi subsp. enterica str. Ty2** | NC 004631.1 |
| **Salmonella serovar Typhi subsp. enterica** | DQ344537.1 |
| **Salmonella serovar Javiana subsp. enterica str. GA MM04042433** | NZ ABEH02000012.1 |
| **Salmonella typhimurium str. S1.I** | DQ153191.1 |
| **Salmonella sp. str. 4064** | FJ405337.1 |
| **Salmonella serovar Newlands subsp. enterica** | DQ344538.1 |
| **Salmonella enteritidis str. E3** | EU118102.1 |
| **Salmonella enterica str. 22/M185/01/98** | FN555100.1 |
| **Salmonella subsp. enterica serovar Dublin str. 57920** | AF227868.1 |
| **Salmonella serovar Choleraesuis subsp. enterica** | DQ344535.1 |
| **Salmonella choleraesuis str. DSM 14846** | EU014681.1 |
| **Salmonella serovar Paratyphi C strain subsp. enterica str.** | CP000857.1 |
| **Salmonella subsp. enterica serovar Typhimurium LT2 str. LT2** | AE006468.1 |
| **Salmonella subsp. enterica serovar Typhimurium str. 65946 subsp.** | AF227869.1 |
| **Salmonella sp. 'group B' HQ010915-1** | EU073022.1 |
| **Salmonella serovar Typhimurium subsp. enterica** | DQ344533.1 |
| **Salmonella sp. str. 4066** | FJ405339.1 |
| **Salmonella serovar Enteritidis subsp. enterica str. ATCC 13076** | EU014687.1 |
| **Salmonella sp. str. 10** | FJ463829.1 |
| **Salmonella enteritidis HQ030906-1** | EU073020.1 |
| **Salmonella sp. str. 4071** | FJ405313.1 |
| **Salmonella sp. str. 2** | FJ463830.1 |
| **Salmonella sp. str. 4072** | FJ405314.1 |
| **Salmonella subsp. enterica serovar Enteritidis str. CQ0709** | FJ465088.1 |
| **Salmonella sp. str. D187-2** | FJ463823.1 |
| **Salmonella enterica str. PRSC-1** | FJ609417.1 |
| **Salmonella serovar Dublin subsp. enterica str. DSPV 595T** | FJ997268.1 |
| **Salmonella serovar Saintpaul subsp. enterica str. SARA23** | NZ ABAM02000001.1 |
| **Shigella boydii** |  |
| **Shigella boydii    3581** | X96965.1 |
| **Shigella boydii str. 3052-94** | AY696681.1 |
| **Shigella boydii str. 3557-77** | AY696660.1 |
| **Shigella boydii str. Sb227** | CP000036.1 |
| **Shigella boydii str. WAB1893** | AM184234.1 |
| **Shigella boydii str. WAB1890** | AM184231.1 |
| **Shigella boydii str. FBD010** | EU009181.1 |
| **Shigella boydii str. FBD009** | EU009180.1 |
| **Shigella boydii str. GTC 779** | AB273731.1 |
| **Shigella boydii str. FBD007** | EU009178.1 |
| **Shigella boydii str. FBD008** | EU009179.1 |
| **Shigella boydii str. Ag07** | EU554433.1 |
| **Shigella dysenteriae** |  |
| **Shigella dysenteriae** | X96966.1 |
| **Shigella dysenteriae** | X80680.1 |
| **Shigella dysenteriae str. Sd197** | CP000034.1 |
| **Shigella dysenteriae str. Sd197** | NC 007606.1 |
| **Shigella dysenteriae str. HBCC-4** | EU409294.1 |
| **Shigella dysenteriae str. 1012** | NZ AAMJ02000006.1 |
| **Shigella dysenteriae str. HDDMN10** | EU881981.1 |
| **Shigella dysenteriae str. 1012** | AAMJ02000006.1 |
| **Shigella dysenteriae str. LS92** | FJ937911.1 |
| **S dysenteriae Sd197** | NC_007606.1 |
| **S dysenteriae GYPB22** | JF346892.1 |
| Shigella flexneri |  |
| **Shigella flexneri** | X96963.1 |
| **Shigella flexneri** | X80679.1 |
| **Shigella flexneri 5 str. 8401** | NC 008258.1 |
| **Shigella flexneri 5 str. 8401** | NC 008258.1 |
| **Shigella flexneri str. FBD004shig** | EF643609.1 |
| **Shigella flexneri str. Ag05** | EU554431.1 |
| **Shigella flexneri** | EU857633.1 |
| **Emergence new multidrug resistance serotype X variant epidemic** | CP001383.1 |
| **Shigella flexneri CCM20B** | FN433027.1 |
| **Shigella flexneri str. A6** | GQ304782.1 |
| **S flexneri 2a str. 301** | NC_004337.2 |
| **S flexneri 5a str. M90T** | NZ_CP037923.1 |
| **Shigella sonnei** |  |
| **Shigella sonnei** | X96964.1 |
| **Shigella sonnei** | X80726.1 |
| **Shigella sonnei str. Ss046** | NC 007384.1 |
| **Shigella sonnei str. Ss046** | NC 007384.1 |
| **Shigella sonnei str. FBD020** | EU009194.1 |
| **Shigella sonnei str. GTC 781** | AB273732.1 |
| **Shigella sonnei str. FBD019** | EU009193.1 |
| **Shigella sonnei str. HDDMN07** | EU881979.1 |
| **Shigella sonnei str. HDDMG06** | EU723822.1 |
| **Shigella sonnei str. 136** | GQ259886.1 |
| **Shigella sonnei 53G** | NC_016822.1 |
| **Vibrio cholerae** |  |
| **Vibrio cholerae str. SIO** | AY494842.1 |
| **Vibrio cholerae str. ATCC 14547** | EU130474.1 |
| **Vibrio cholerae str. 623-39** | AAWG01000229.1 |
| **Vibrio cholerae str. asiaticus ATCC14035** | Z21856.1 |
| **Vibrio cholerae** | AY292952.1 |
| **Vibrio cholerae RC356 RC725** | EF684905.1 |
| **Vibrio cholerae RC395** | EF684904.1 |
| **Vibrio cholerae RC386** | EF684901.1 |
| **Vibrio cholerae bv. albensis str. ATCC 14547 RC782** | EF032499.1 |
| **Vibrio cholerae RC466** | EF684900.1 |
| **Vibrio cholerae str. MJ-1236** | CP001485.1 |
| **Vibrio cholerae str. O395** | NC 012582.1 |
| **Vibrio cholerae str. ATCC 14033** | X74694.1 |
| **Vibrio cholerae str. VC12-Ogawa** | AY513500.1 |
| **Vibrio cholerae str. TP** | AY494843.1 |
| **Vibrio cholerae str. ATCC 14035 RC2** | EF032498.1 |
| **Vibrio cholerae str. 2740-80** | AAUT01000059.1 |
| **Vibrio cholerae str. O395** | NC 009457.1 |
| **Vibrio cholerae str. MZO-2** | NZ AAWF01000074.1 |
| **Vibrio cholerae str. CECT 514 T** | X76337.1 |
| **Vibrio cholerae RC483** | EF684899.1 |
| **Vibrio cholerae str. MJ-1236** | CP001485.1 |
| **Vibrio cholerae str. UN 13140** | DQ068935.1 |
| **Vibrio cholerae str. LD081008B-1** | GQ205447.1 |
| **Yersinia pestis** |  |
| **Yersinia pestis biovar Orientalis str. MG05-1020** | NZ AAYS01000007.1 |
| **Yersinia pestis biovar Orientalis str. MG05-1020** | NZ AAYS01000002.1 |
| **Yersinia pestis biovar Orientalis str. F1991016** | ABAT01000007.1 |
| **Yersinia pestis SS-Yp-106** | AJ232230.1 |
| **Yersinia pestis str. Antiqua** | NC 008150.1 |
| **Yersinia pestis str. Antiqua** | CP000308.1 |
| **Yersinia pestis str. Pestoides F** | NC 009381.1 |
| **Yersinia pestis str. CA88-4125** | NZ ABCD01000004.1 |
| **Yersinia pestis str. Angola** | NC 010159.1 |
| **Yersinia pestis str. CA88-4125** | NZ ABCD01000006.1 |
| **Yersinia pestis biovar Antiqua str. E1979001** | NZ AAYV01000028.1 |
| **Yersinia pestis biovar Antiqua str. B42003004** | NZ AAYU01000002.1 |
| **Yersinia pestis biovar Orientalis str. F1991016** | NZ ABAT01000001.1 |
| **Yersinia pestis biovar Orientalis str. IP275** | AAOS02000054.1 |
| **Yersinia pestis biovar Antiqua str. UG05-0454** | NZ AAYR01000040.1 |
| **Yersinia pestis str. Nepal516** | NC 008149.1 |
| **Yersinia pestis str. Pestoides F** | CP000668.1 |
| **Yersinia pestis str. CA88-4125** | ABCD01000005.1 |
| **Yersinia pestis biovar Antiqua str. B42003004** | NZ AAYU01000008.1 |
| **Yersinia pestis biovar Antiqua str. E1979001** | NZ AAYV01000004.1 |
| **Yersinia pestis biovar Antiqua str. UG05-0454** | AAYR01000007.1 |
| **Yersinia pestis biovar Orientalis str. MG05-1020** | AAYS01000005.1 |
| **Yersinia pestis biovar Mediaevalis str. 1973002** | AAYT01000010.1 |
| **Yersinia pestis biovar Antiqua str. B42003004** | NZ AAYU01000012.1 |
| **Yersinia pestis str. D182038** | CP001589.1 |
| **Yersinia pestis str. IM** | AE013647.1 |
| **Yersinia pestis str. CO92** | AJ414158.1 |
