# Supplementary material for: Evaluation of 16S rRNA Hypervariable Regions for Bioweapon Species Detection by Massively Parallel Sequencing
Source: Int J Microbiol. 2020 Sep 26;2020:8865520. doi: 10.1155/2020/8865520 (PMC7533751; doi:10.1155/2020/8865520)
Supplement: Supplementary Materials — Table S1: 16S rRNA gene sequences used as a source for the selection of variable regions from each of the 19 selected strains of bacterial species. Table S2: bacterial DNA source for the simulated metagenomic community. Figure S1: alignment of the consensus sequences of the 16S rRNA gene. The conserved regions are indicated by a color scale, from green (more conserved) to yellow (less conserved), and the most varied or gaps are represented in white. The blue rectangles correspond to the identified hypervariable regions of the alignment. Figure S2: the 16S rRNA gene locus and location of hypervariable regions and primers developed in this study. Blue rectangles represent the hypervariable regions V1–V9; the forward primers are indicated in dark green and the reverse primers in light green. Table S3: source of samples, target region of 16S rRNA gene primers, and sequencing platform used in the study experiments. Table S4: percentage mapped and unmapped reads of the bacterial mock community sequenced in Illumina MiSeq platform. Reads discriminated using the primer regions, tested individually in two replicates, and denominated by the suffixes I and II. Figure S3: taxonomic bacterial classification of an environmental sample sequenced in Illumina MiSeq platform. Distribution determined using combinations of primer sets targeting 16S rRNA gene hypervariable regions (V1-V2-V4-V5-V6-V7-V8). Classifications were obtained using Kraken. Table S5: sensitivity of the primer sets to detect a diverse group of bacteria in a human DNA background to simulate infected subjects. Figure S4: number of reads mapped to the consensus 16S rRNA in an environmental sample without addition of mock bacterial community (0 GE) and with simulated bacterial scenarios sequenced in Illumina MiSeq platform. The most accurate combinations of primer sets targeting 16S rRNA gene hypervariable regions (V1-V2-V4-V5-V6-V7-V8) are revealed by mapping reads to consensus 16S rRNA using only Bowtie2. The 2,000 [file 8865520.f1.zip › 8865520.f1/Table S4.docx]

Table S4: Percentage of mapped and unmapped reads on the bacterial mock, discriminated by the primers regions, tested individually, in two replicas denominated by the suffix I and II)

| **Sample** | **V1V2_I** | **V1V2_II** | **V4V5_I** | **V4V5_II** | **V6V7V8_I** | **V6V7V8_II** |
| --- | --- | --- | --- | --- | --- | --- |
| **Total** **reads** | 1044000 | 496560 | 556082 | 403818 | 536186 | 646544 |
| **After Trimming (0.05)** | 1022000 | 479450 | 545660 | 398862 | 528585 | 635678 |
| **Avg length (bp)** | 250 | 250 | 249 | 250 | 250 | 250 |
| Mapped reads | 910169 | 437619 | 511689 | 377962 | 499690 | 602354 |
| Mapped reads (%) | 89,06% | 91,28% | 93,77% | 94,76% | 94,53% | 94,76% |
| Unmapped reads | 111831 | 41831 | 33971 | 20900 | 28895 | 33324 |
| Unmapped reads (%) | 10,94% | 8,72% | 6,23% | 5,24% | 5,47% | 5,24% |
